# Supplementary material for: Hot spot prediction in protein-protein interactions by an ensemble system
Source: BMC Syst Biol. 2018 Dec 31;12(Suppl 9):132. doi: 10.1186/s12918-018-0665-8 (PMC6311905; doi:10.1186/s12918-018-0665-8)
Supplement: Supplementary file 2 — All relevant MATLAB code. A simple Matlab implement of our predictor. (ZIP 19,742 kb) [file 12918_2018_665_MOESM2_ESM.zip › Additional file 2/Read ASA/ASA website/dbMPIKT/1KN0A_files/title_entry_3.html]

阅读模式-气泡

不再提示

 

###### 进入阅读模式纯净浏览

今日已有 *- - 0
  - 1
  - 2
  - 3
  - 4
  - 5
  - 6
  - 7
  - 8
  - 9
- - 0
  - 1
  - 2
  - 3
  - 4
  - 5
  - 6
  - 7
  - 8
  - 9
- - 0
  - 1
  - 2
  - 3
  - 4
  - 5
  - 6
  - 7
  - 8
  - 9
- - 0
  - 1
  - 2
  - 3
  - 4
  - 5
  - 6
  - 7
  - 8
  - 9
- - 0
  - 1
  - 2
  - 3
  - 4
  - 5
  - 6
  - 7
  - 8
  - 9
- - 0
  - 1
  - 2
  - 3
  - 4
  - 5
  - 6
  - 7
  - 8
  - 9* 人使用

立即进入
